# Three Cases of Anaplastic Thyroid Carcinoma Transformation and Leukocytosis during Lenvatinib Treatment

**DOI:** 10.1155/2020/6667237

**Published:** 2020-11-09

**Authors:** Hiroyuki Iwasaki, Soji Toda, Daisuke Murayama, Hiroyuki Takahashi

**Affiliations:** ^1^Department of Breast and Endocrine Surgery, Kanagawa Cancer Center, Yokohama, Japan; ^2^Department of Oncology, Kanagawa Cancer Center, Yokohama, Japan

## Abstract

Since 2015, the cancer treatment lenvatinib has been used for patients with advanced radioactive iodine- (RAI-) refractory thyroid differentiated cancer; however, the drug's long-term effects have not been fully investigated. We report three cases in which lenvatinib treatment initially improved the patients' conditions, although they all died approximately 2 months after leukocytosis due to very aggressive disease progression with anaplastic thyroid carcinoma transformation. Serum interleukin-6 (IL-6) was elevated in all three cases, and granulocyte-colony stimulating factor (G-CSF) was elevated in two cases. The patients had a similar clinical course, with multiorgan metastasis and aggressive disease progression. Even with advanced cancer, lenvatinib has provided control of the disease. However, as long-term use of lenvatinib grows, it is possible that similar cases will increase, and we report our findings as an alert to other clinicians.

## 1. Introduction

Lenvatinib has been recognized to be effective against progressive radioactive iodine- (RAI-) refractory differentiated thyroid carcinoma (DTC) [1]. This targeted cancer drug has been used in Japan with documented effectiveness since 2015 [2]. We saw three patients who underwent lenvatinib treatment and died approximately 2 months after anaplastic transformation with leukocytosis. They initially saw good results and shrinkage of their tumors from the lenvatinib use, but they developed excessive leukocytosis and rare anaplastic thyroid cancer. There have been other cases where patients who were later diagnosed with anaplastic thyroid cancer (ATC) originally presented with excessive leukocytosis and high levels of granulocyte-colony stimulating factor (G-CSF) and interleukin-6 (IL-6) [3]. Very rarely, leukocyte hyperplasia and rapid disease progression have been reported as aggressive cancers in the lung or stomach [4, 5]. In addition to describing our patients' experience with this aggressive disease, we will caution other clinicians about the possible consequences of long-term lenvatinib treatment.

## 2. Case Presentation

The following three patients were initially diagnosed with DTC and treated at the Kanagawa Cancer Center, Japan. During the postoperative follow-up, disease progression with distant metastases was observed, followed by treatment with lenvatinib. Although the treatment process was satisfactory, anaplastic transformation developed unexpectedly with leukocytosis and aggressive disease progression. This study was approved by the Institutional Review Board of Kanagawa Cancer Center (IRB approval number 27–61 for DTC and 28–49 for ATC). All patients provided a comprehensive consent stating that their samples collected for medical examination can be utilized for investigation and clinical research.

Case 1 was a 58-year-old woman with an unexceptional medical history (height: 152 cm and weight: 42 kg). She had been diagnosed with thyroid cancer that metastasized to the lungs and pelvis; she had previously undergone total thyroidectomy in 2015. The pathology was follicular thyroid carcinoma containing poorly differentiated components, and she was treated postoperatively by external beam radiation to the left pelvis, denosumab, and three courses of RAI treatment. The pelvic metastasis remained unchanged, the pulmonary metastasis was exacerbated, and her serum thyroglobulin (Tg) level gradually increased in 5 years after the initial surgery. Then, we started treatment with lenvatinib at an initial dose of 14 mg, decreased to 10 mg after eight weeks, and the adverse events (AE) included grade 1 hypertension (HT) and grade 1 appetite loss. The pulmonary metastasis decreased slightly, but the pelvic metastasis remained unchanged; the efficacy according to the response evaluation criteria in solid tumors (RECIST) was stable disease [6]. Twelve weeks after commencing lenvatinib treatment, she developed a high fever with excessive leukocytosis (white blood cells (WBC) = 50300/*μ*L).

**Figure 1:**
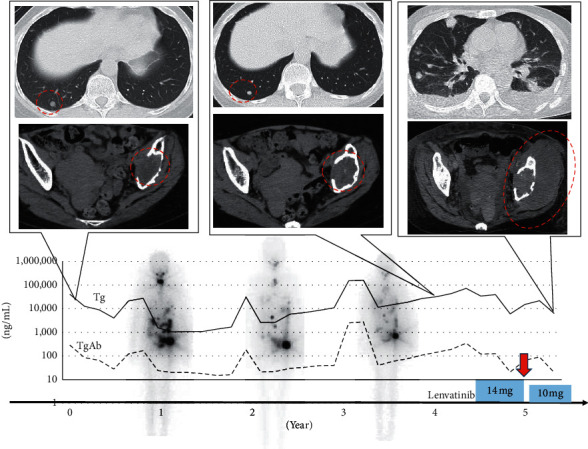
Clinical course of Case 1. The upper image is a chest computed tomography (CT) image. Although lung metastasis did not change after three courses of radioactive iodine (RAI) treatment, the patient's condition became progressive disease after anaplastic transformation and bilateral pleural effusion accumulated. The lower image is a CT image of the pelvis, and the left iliac metastasis increased rapidly after anaplastic transformation. The graph below the image shows the transition of thyroglobulin and antibodies, the timing of lenvatinib treatment and the dose. Whole body scintigraphy images after three RAI treatments are also shown in the background. RAI uptake is decaying. The red dotted circle indicates the lesion. The red arrow indicates the time of anaplastic transformation.

When the lenvatinib treatment was discontinued, her WBC increased to 70,800 *μ*L, followed by an increase in G-CSF to 748 pg/mL and IL-6 to 114 pg/mL; there was a continued progression of the pulmonary metastasis, pleural effusion, and a rapid increase in the necrosis of the left pelvic metastasis (Figure 1). Drainage was performed, and cytology was submitted twice, but it was negative. The patient transitioned to best supportive care (BSC) and died 51 days later. The duration of lenvatinib treatment was 5.1 months.

Case 2 was a 70-year-old woman with an unexceptional medical history (height: 157 cm and weight: 50 kg). She had surgery in 2004 previously for papillary thyroid carcinoma (PTC), and multiple pulmonary metastases were recognized in 2009. RAI treatment was performed twice but was ineffective for the pulmonary metastasis, which gradually increased. Active surveillance continued with thyroid stimulating hormone (TSH) suppression therapy, but the pulmonary tumor increased to 17 mm in 2015. We started lenvatinib treatment at the initial dose of 24 mg, which was reduced to 10 mg after 12 weeks. The AE induced by lenvatinib included grade 3 HT, grade 1 alopecia, and grade 2 arthralgia.

**Figure 2:**
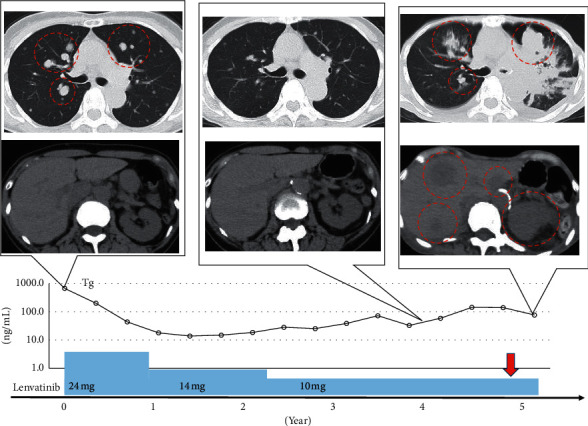
Clinical course of Case 2. The upper image is a chest computed tomography (CT) image. The patient was classified as partial response after treatment with lenvatinib, but then was reclassified as progressive disease after anaplastic transformation and became left atelectasis. The lower image is a CT image of the upper abdomen, and multiple liver metastases and metastases to the left adrenal gland and kidney appeared after anaplastic transformation. The graph below the image shows the transition of thyroglobulin, the timing of lenvatinib treatment and the dose. The red dotted circle indicates the lesion. The red arrow indicates the time of anaplastic transformation.

The tumor shrank upon treatment, from 17 to 9 mm in diameter, and the RECIST efficacy was categorized as a partial response. While the disease condition was stable for 56 months following lenvatinib treatment, her WBC unexpectedly increased to 19,700, and a computed tomography (CT) scan showed exacerbation of the pulmonary metastasis, left lung S3 atelectasis, and pleural effusion, which gave a negative cytology result; she was reclassified with progressive disease. Her WBC continued to increase to 33,700, G-CSF was 22.4 pg/mL, within a normal range, but IL-6 was elevated to 22.8 pg/mL. When her physical condition began to deteriorate, with weight loss and an inability to walk, a CT scan revealed multiple metastases on the liver, left adrenal gland, and kidneys (Figure 2). She transitioned to BSC and died 50 days after the rise in WBC. The duration of lenvatinib treatment was 5.09 years.

Case 3 was a 71-year-old woman with a history of HT, hyperlipidemia, and chronic kidney disease (height: 154 cm and weight: 73 kg). She was diagnosed with PTC, and her initial surgery was performed in 2002. Multiple pulmonary metastases had been recognized in 2011, but RAI treatment was not effective. The maximum diameter of pulmonary metastasis was 6 mm, the serum Tg level remained stable at <1 ng/dL for 8 years after the diagnosis of pulmonary metastasis, and active surveillance was continued with TSH suppression; however, the size of the pulmonary tumor increased to 15 mm in 2019.

**Figure 3:**
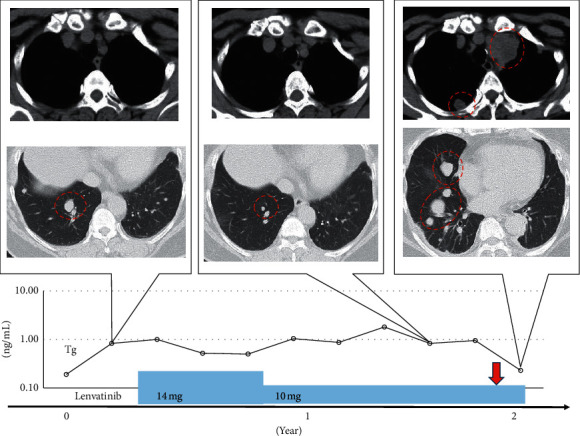
Clinical course of Case 3. The upper image is a chest computed tomography image of the mediastinal condition, and new lesions appeared in the mediastinal lymph nodes and pleura. In the lower image, the target lesion became partial response after lenvatinib treatment; then, it became progressive disease after anaplastic transformation. The graph below the image shows the transition of thyroglobulin, the timing of lenvatinib treatment, and the dose. The red dotted circle indicates the lesion. The red arrow indicates the time of anaplastic transformation.

We started lenvatinib treatment at the initial dose of 14 mg, which was reduced to 10 mg after four weeks. The AE induced by lenvatinib included grade 2 HT and grade 1 stomatitis. The tumor shrank from 15 mm to 6 mm in diameter, and the efficacy was categorized as a partial response. Ten months after commencing lenvatinib treatment, her WBC increased to 19,700, and a CT scan showed multiple new lesions of 28 mm, changing her disease category to progressive disease (Figure 3). Her WBC increased to 33,700, G-CSF to 617 pg/mL, and IL-6 to 46.4 pg/mL. She developed low back pain, and an examination revealed progressive pulmonary and pelvic bone metastases. She was given 20 Gy external beam radiation to treat bone metastasis but suffered weight loss. She transitioned to BSC, and she died 66 days after the initial rise in WBC. The duration of lenvatinib treatment was 13.5 months.

## 3. Discussion

### 3.1. Characteristics of Anaplastic Thyroid Carcinoma Transformation

ATC is one of the most aggressive neoplasms. There have been several reports of leukocytosis due to the leukemoid paraneoplastic reaction resulting from G-CSF, granulocyte-macrophage (GM) CSF, and IL-6 production. Excessive WBC counts that continue to increase are linked to a poorer prognosis [7–11]. However, there are very few reports where leukocytosis is a primary marker in an ATC diagnosis. Since 1992, 17 cases diagnosed as ATC with leukocytosis have been reported worldwide, of which 13 cases have been in Japan [3]. It is significant that three patients in the same facility were diagnosed within 3 months for this disease. The clinical course of each case was similar, and it is especially notable that each treatment course was controlled by the use of lenvatinib.

### 3.2. Blood Results

**Figure 4:**
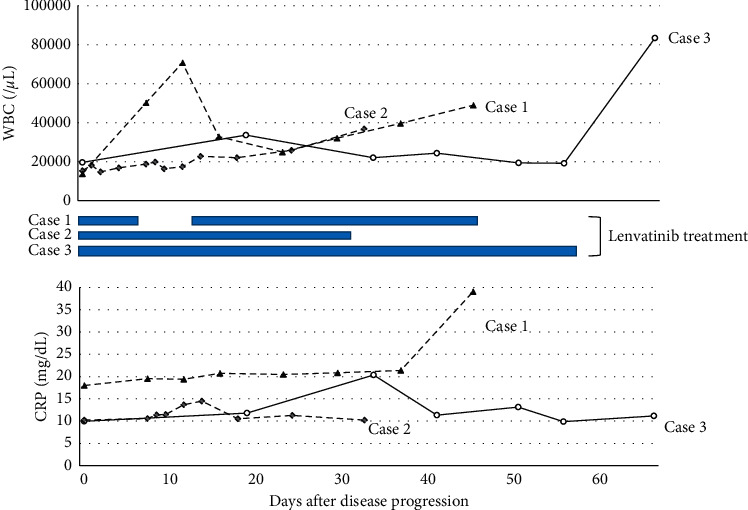
Changes in white blood cell counts and C-reactive protein. This figure shows changes after leukocytosis was observed in three cases. Leukocytes in Case 1 increased rapidly with a one-week washout of lenvatinib, decreased with resumption, but increased again. Patients in the second and third cases did not stop taking lenvatinib, based on the experience of Case 1. The broken line indicates Case 1, the dotted line indicates Case 2, and the solid line indicates Case 3.

Table 1 shows inflammatory reactions demonstrated by increased WBCs, hemoglobin, platelet counts, neutrophil-lymphocyte ratio, C-reactive protein (CRP), G-CSF, and IL-6 in all cases.  Figure 4 shows the WBC counts, which remained high in all patients, although the CRP did not decrease. The high fever observed in cases 1 and 2 was successfully treated with naproxen, but the leukocytosis did not subside. Lenvatinib was continued. After the WBC increased, the disease progressed rapidly, and all of them died approximately 2 months later. The serum Tg levels for each patient are shown in Figures 1–3, but Tg levels cannot be used as an indicator in ATC; so, the disease progression and the increase in Tg were not linked.

### 3.3. Analysis of G-CSF and IL-6 and Cancer

G-CSF is a growth factor that is known to be a key regulator of hemopoiesis and for the proliferation and differentiation of neutrophils. G-CSF-producing tumors have aggressive characteristics that have been the subject of many Japanese investigations [4, 5, 12]. G-CSF-producing malignant tumors are sometimes accompanied by fever and high CRP levels, but G-CSF itself does not have the effect of inducing these reactions, and an inflammatory cytokine such as IL-6 could be involved. IL-6 is secreted by T lymphocytes; it differentiates B lymphocytes into antibody-producing cells and is an acute phase response during inflammation [13].

### 3.4. Future Monitoring and Investigation

In the future, if a patient experiences progressive disease while being treated with lenvatinib, the clinician should investigate whether anaplastic transformation has developed. Since there are limited long-term results for lenvatinib treatment alone, genetic tests should be performed, and if indicated, BRAF inhibitors and PD-L1 inhibitors should be used in combination; however, these are not yet available for clinical use in Japan [14]. Until then, clinicians should monitor the clinical course and leukocyte levels of patients being treated with lenvatinib. The findings from our institution also warrant further research from other investigators whose patients have had a similar clinical course.

## Figures and Tables

**Table 1:** Blood test findings in the three cases.

Case	1	2	3
WBC ( /*μ*L)	70800	36900	83500
Hb (g/dL)	8.4	11.5	10.6
Neutrophils (%)	86	95	92
Lymphocytes (%)	3	2	7
Eosinophils (%)	4		
Monocytes (%)	2	2	1
Myelocytes (%)	3		
NLR	28.7	47.5	13.1
PLT(104/*μ*L)	31.6	17.8	29.4
CRP(mg/dL)	19.38	10.24	11.16
G-CSF(pg/mL)	748	22.4	617
IL-6 (pg/mL)	114	22.8	46.4

NLR, neutrophil/lymphocyte ratio; CRP, C-reactive protein; G-CSF, granulocyte-colony stimulating factor; IL-6, interleukin-6.The normal value of G-CSF is 5.78–27.5 pg/mL, and the normal value of IL-6 is 8 pg/mL or less.

## Data Availability

The datasets used and/or analyzed during the current study are available from the corresponding author upon request.
